# Kelp Culture Enhances Coastal Biogeochemical Cycles by Maintaining Bacterioplankton Richness and Regulating Its Interactions

**DOI:** 10.1128/msystems.00002-23

**Published:** 2023-02-16

**Authors:** Yi Sun, Hongjun Li, Xiaocheng Wang, Hongbo Li, Ye Deng

**Affiliations:** a State Environmental Protection Key Laboratory of Coastal Ecosystem, National Marine Environmental Monitoring Center, Dalian, China; b College of Resources and Environment, University of Chinese Academy of Sciences, Beijing, China; Max Planck Institute for Marine Microbiology

**Keywords:** kelp culture, bacterioplankton, biogeochemical cycling, co-occurrence network, biodiversity and ecosystem function

## Abstract

As an important carbon sink, seaweed cultivation plays a vital role in controlling global climate change. However, most studies have been focused on the seaweed itself, and knowledge of bacterioplankton dynamics in seaweed cultivation activities is still limited. Here, a total of 80 water samples were obtained from a coastal kelp cultivation area and adjacent non-culture area in the seedling and mature stages. The bacterioplankton communities were analyzed using high-throughput sequencing of bacterial 16S rRNA genes, and the microbial genes involving biogeochemical cycles were measured by a high-throughput quantitative PCR (qPCR) chip. Seasonal variations in alpha diversity indices of bacterioplankton were found, and kelp cultivation mitigated this decline in biodiversity from the seedling to the mature stage. Further beta diversity and core taxa analyses revealed that the maintenance of biodiversity was due to kelp cultivation favoring the survival of rare bacteria. Comparisons of gene abundances between coastal water with and without kelp cultivation showed a more powerful capacity of biogeochemical cycles induced by kelp cultivation. More importantly, a positive relationship between bacterial richness and biogeochemical cycling functions was observed in samples with kelp cultivation. Finally, a co-occurrence network and pathway model indicated that the higher bacterioplankton biodiversity in kelp culture areas compared to non-mariculture regions could balance the microbial interactions to regulate biogeochemical cycles and thus enhance the ecosystem functions of kelp cultivation coasts. The findings of this study allow us to better understand the effects of kelp cultivation on coastal ecosystems and provide novel insights into the relationship between biodiversity and ecosystem functions.

**IMPORTANCE** In this study, we tried to address the effects of seaweed cultivation on the microbial biogeochemical cycles and the underlying relationships between biodiversity and ecosystem functions. We revealed clear enhancement of biogeochemical cycles in the seaweed cultivation areas compared to the non-mariculture coasts at both the beginning and ending of the culture cycle. Moreover, the enhanced biogeochemical cycling functions in the culture areas were found to contribute to the richness and interspecies interactions of bacterioplankton communities. The findings of this study allow us to better understand the effects of seaweed cultivation on coastal ecosystems and provide novel insights into the relationship between biodiversity and ecosystem functions.

## INTRODUCTION

Coastal ecosystems are bridges between the land and ocean, which is the most active area for various material transformation and energy flow (1). Coastal ecosystems contribute more than 25% of global primary production (2), in which microorganisms are the main drivers of the biogeochemical cycles of important elements, such as carbon, nitrogen, sulfur, and phosphorus (3). With increasing global climate change, carbon sequestration in coastal ecosystems is increasingly important to inhibit global warming (4). In addition, numerous anthropogenic activities occurring in coastal ecosystems can also influence the biogeochemical cycles of microbes, thereby affecting coastal ecosystem functions (5, 6). Unlike most anthropogenic activities which are seen as “carbon sources,” seaweed cultivation has received attention as a possible “carbon sink” (7). Beyond the photosynthesis of seaweed itself, the carbon sequestration by microorganisms in the culture area through biogeochemical cycles cannot be ignored (8).

Microbial communities in seaweed cultivation zone are highly complex, composed of eukaryotes, bacteria, archaea, and viruses (9). Among these, bacteria are the dominant component of this ecosystem and play an important role in biogeochemical cycles (10). Previously, most research focusing on the microorganisms associated with seaweed followed interest in isolating bacterial strains to degrade fibers (11–13). Recently, some studies have considered the effects of seaweed cultivation on microbial communities in culture areas. For example, differences in water and sediment microbial communities between seaweed cultivation zones and non-culture zones have been observed on the coast of Nan’ao Island, China (14). The profiles of microbial communities in seawater during the entire process of seaweed cultivation in coast of Dalian, China, have also been uncovered (15). Moreover, the functional roles of seaweed-associated microbiomes were explored in an investigation of metagenome-assembled genomes (16). However, there is no clear understanding of the effects of seaweed cultivation on the biogeochemical cycles of microbial communities and their underlying mechanisms.

Diverse organisms in ecosystems comprise a large amount of biodiversity and are responsible for multiple ecosystem functions (17). Reduction in ecosystem functions due to biodiversity loss has been uncovered by many experiments, highlighting the importance of biodiversity for maintaining ecosystem functions (18). In recent years, studies on the relationships between biodiversity and ecosystem functioning (B-EF) have increased substantially, showing the positive B-EF relationships in diverse ecosystems (19–21). Unlike macrofauna which directly perform ecosystem functions, microorganisms have huge diversity and develop complex interactions to maintain their ecological stability (22). Microbial networks are frequently used to investigate the interspecific interactions of the microbial community, in which nodes represent species and edges represent interactions between species (23). In a microbial network, nodes which are strongly connected with each other are compartmentalized into a module, which can be considered an indicator for different niches and ecosystem functions (24). The microbial module is a distinction and clustering of biodiversity, which could explain the underlying mechanisms of B-EF relationships (25). Although several investigations of B-EF relationships have been reported, our understanding of how seaweed cultivation influences B-EF relationships related to microbes is still limited.

During seaweed cultivation, high seaweed density provides more dissolved oxygen and nutrients in the culture zone (15). Appropriate conditions may promote the development of bacterioplankton communities in seaweed culture areas (26). Thus, we proposed a hypothesis that seaweed cultivation could be beneficial to the biodiversity of the bacterioplankton community in the culture area, thereby enhancing the ecosystem functions related to them. To test this hypothesis, we obtained water samples from a kelp culture area and an adjacent non-culture area during the seedling and mature stages. Bacterioplankton communities were investigated using high-throughput (HT) sequencing based on the bacterial 16S rRNA gene, and the genes of microbial biogeochemical cycles were measured by a HT-qPCR (quantitative PCR) chip. Our findings will enhance our understanding of carbon cycles related to bacterioplankton in kelp cultivation ecosystems and provide novel insights into the B-EF relationships.

## RESULTS AND DISCUSSION

### Kelp cultivation maintains bacterioplankton biodiversity.

In this study, a total of 80 water samples were obtained from a representative seaweed culture and adjacent non-culture control areas in the seedling and mature stages. The bacterioplankton from these water samples were investigated by Illumina sequencing based on bacterial 16S rRNA genes. Overall, 6,612,633 high-quality sequences (average of 66,082, 72,982, 97,871, and 93,697 sequences for MW.S [mariculture water seedling], CW.S [control water seedling], MW.M [MW mature], and CW.M [CW mature] samples, respectively) were retrieved (Table S1), which were clustered into 9,500 amplicon sequence variants (ASVs). According to taxonomic annotation results, 99.57% of the ASVs were successfully assigned to a bacterial phylum, and 73.83% of ASVs were assigned at the genus level (Fig. S1). In total, 32 phyla, 67 classes, 183 orders, 335 families, and 740 genera were annotated from these ASVs (Table S2). Rarefaction curves for all samples were close to the horizontal state (Fig. S2), indicating that the sequencing depth was sufficient to reflect the richness of each bacterioplankton community analyzed.

10.1128/msystems.00002-23.1 FIG S1 Annotation of taxonomy at different levels. Download FIG S1, PDF file, 0.01 MB. Copyright © 2023 Sun et al. 2023 Sun et al. https://creativecommons.org/licenses/by/4.0/ This content is distributed under the terms of the Creative Commons Attribution 4.0 International license.

10.1128/msystems.00002-23.2 FIG S2 Rarefaction curves. Download FIG S2, PDF file, 0.02 MB. Copyright © 2023 Sun et al. 2023 Sun et al. https://creativecommons.org/licenses/by/4.0/ This content is distributed under the terms of the Creative Commons Attribution 4.0 International license.

10.1128/msystems.00002-23.6 TABLE S1 Statistics of sequencing data. Download Table S1, DOCX file, 0.02 MB. Copyright © 2023 Sun et al. 2023 Sun et al. https://creativecommons.org/licenses/by/4.0/ This content is distributed under the terms of the Creative Commons Attribution 4.0 International license.

10.1128/msystems.00002-23.7 TABLE S2 Statistics of taxonomic annotation. Download Table S2, DOCX file, 0.02 MB. Copyright © 2023 Sun et al. 2023 Sun et al. https://creativecommons.org/licenses/by/4.0/ This content is distributed under the terms of the Creative Commons Attribution 4.0 International license.

**FIG 1:**
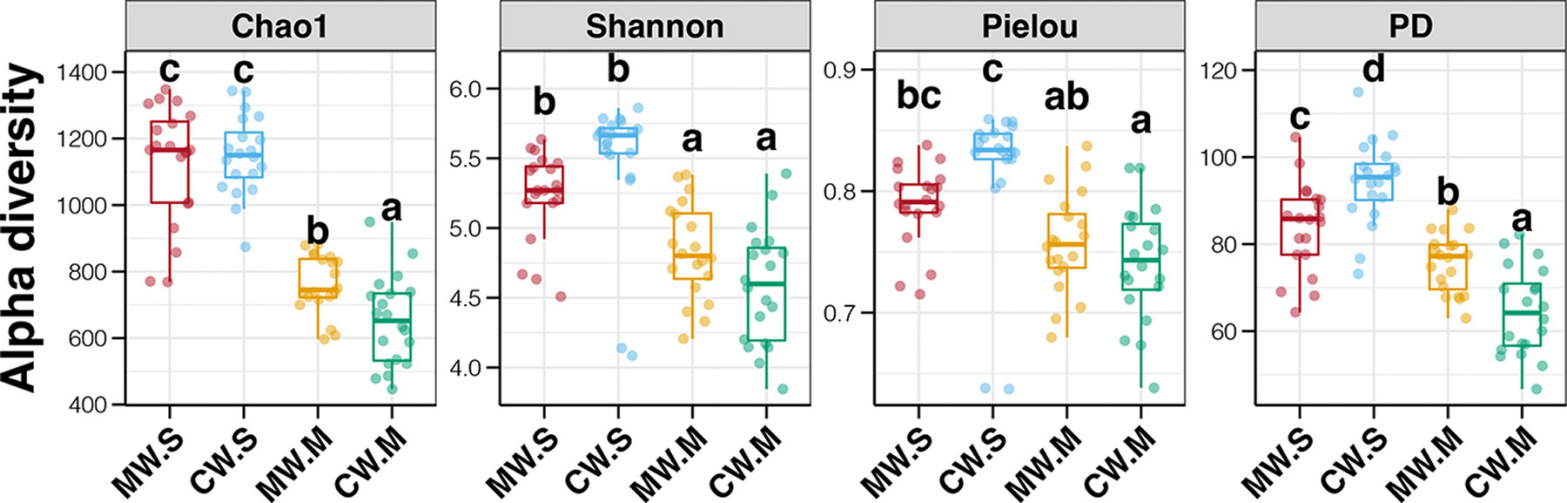
Differences in the alpha diversity indices of bacterioplankton communities among the mariculture water (MW) and control water (CW) samples from the seedling and mature stages. Different lowercase letters above each box in the same figure panel represent significant differences between groups (Tukey’s honestly significant difference [HSD] test, *P* < 0.05). S and M in the group labels represent the seedling and mature stages, respectively.

Four alpha diversity indices of these bacterioplankton communities were calculated and compared, as shown in Fig. 1. In the mature stage, all four alpha diversity indices of bacterioplankton communities were significantly lower than those at the seedling stage for both MW and CW samples (Tukey’s honestly significant difference [HSD] test, *P < *0.05). These obvious declines in bacterioplankton alpha diversity could be due to seasonal variations in environmental conditions (27). The water temperature in the mature stage was significantly lower than that in the seedling stage (Student’s *t* test, *P < *0.05, Fig. S3). While it was a bit strange that the water temperature in December (seedling stage) was higher than that in May (mature stage), this is normal for the study region. Remarkably lower alpha diversities of bacterioplankton from river, estuary, marine, and coastal ecosystems in low-temperature regions have been observed in multiple previous studies (15, 28–30). In addition, consistent changes in bacterioplankton alpha diversity in both MW and CW samples indicated that season may be a stronger factor than kelp culture activities in affecting bacterioplankton dynamics.

10.1128/msystems.00002-23.3 FIG S3 Differences in water temperature between seedling and mature stages. Download FIG S3, PDF file, 0.01 MB. Copyright © 2023 Sun et al. 2023 Sun et al. https://creativecommons.org/licenses/by/4.0/ This content is distributed under the terms of the Creative Commons Attribution 4.0 International license.

Differences in bacterioplankton alpha diversity between MW and CW samples at each culture site were further investigated (Fig. 1). No significant differences in the Chao1 index were found between samples from MW and CW groups at the seedling stage (Tukey’s HSD test, *P > *0.05); however, significant higher values were observed in MW samples compared to CW samples at the mature stage (*P < *0.05). Meanwhile, the phylogenetic diversity (PD) index of bacterioplankton in MW samples was significantly lower than that in CW samples at the seedling stage (*P < *0.05); in contrast, this index was significantly higher in MW samples at the mature stage (*P < *0.05). In addition, the Shannon and Peilou indices of MW and CW samples were not significantly different within a single stage (*P > *0.05).

A steady decline in bacterioplankton diversity during culture progression was found in a previous study of a culture of the seaweed Undaria pinnatifida (15). This decrease in biodiversity might be due to the selectivity for specific microbes with seaweed growth and changes in environmental parameters in the culture area (31). Although there have been no other reports comparing bacterioplankton communities in cultured and non-cultured areas covering the full seaweed culture cycle, some studies have demonstrated greater bacterioplankton biodiversity in seawater with seaweed cultivation compared to water without cultivation in the mature stage (14, 32). The higher biodiversity of bacterioplankton in seawater with high-density seaweeds could attributed to the seaweed-microbial interactions and low solar radiation in this area (33, 34). More importantly, according to our results, the declines in all indices from the seedling to mature stages were obviously lower in the MW group compared to those in CW samples (Fig. 1a). Taken together, the results of previous and current studies indicate that kelp cultivation is conductive to maintaining bacterioplankton biodiversity in culture areas during seasonal variation.

### Biodiversity of bacterioplankton is contributed by low-abundance uncommon ASVs.

**FIG 2:**
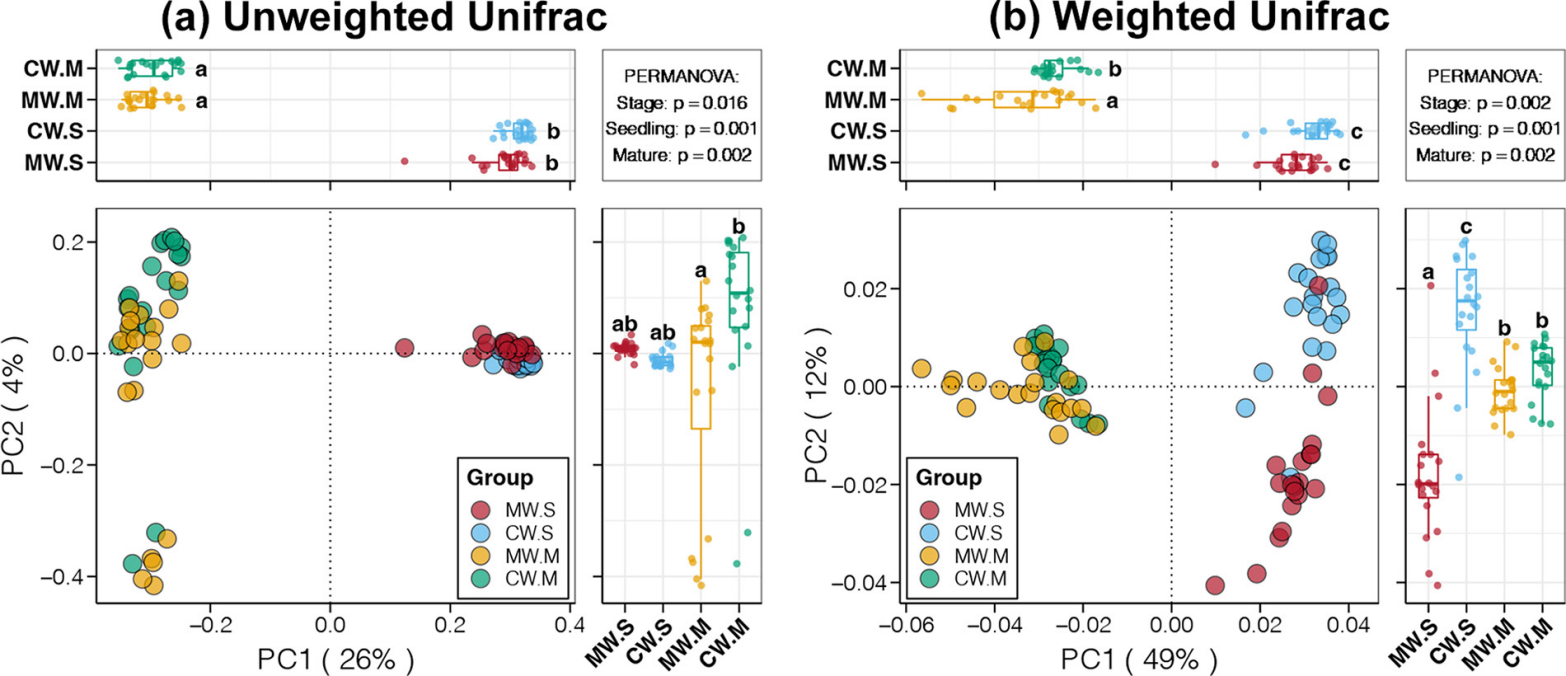
Principal-coordinate analysis (PCoA) and permutational multivariate analysis of variance (PERMANOVA) of bacterioplankton communities among the MW and CW samples from the seedling and mature stages based on (a) unweighted and (b) weighted Unifrac distances, respectively. Different lowercase letters above each box in the same panel represent significant differences between groups (Tukey’s HSD test, *P* < 0.05).

Both unweighted and weighted Unifrac distances were performed to evaluate bacterioplankton composition variations induced by kelp culture. Stage variations in the bacterioplankton compositions were first confirmed by principal-coordinate analysis (PCoA) and permutational multivariate analysis of variance (PERMANOVA) based on both unweighted and weighted Unifrac distances (*P < *0.05, Fig. 2). This phenomenon might come from natural seasonal changes, may be stronger than effects of kelp cultivation, and likely affects alpha diversity. In addition, the unweighted Unifrac distance values were remarkably higher than the weighted distance values (Fig. 2). Unifrac distance introduces evolutionary differences of bacteria into calculation and only considers the species present in the unweighted method, while both the species present and their relative abundances are considered in the weighted method (35). This finding suggested that variations in bacterioplankton were mostly contributed by biodiversity and not by bacterial relative abundances.

For the bacterioplankton in each culture stage, significant differences were found in the mature stage but not in the seedling stage based on the unweighted Unifrac distance (Tukey’s HSD test, *P < *0.05, Fig. 2a). In contrast, the exact opposite results were obtained by weighted Unifrac distance (Fig. 2b). At the seedling stage, we needed to put a lot of seaweed seedlings into the culture area, which could have introduced some foreign bacteria attached to the seedlings or from operating machines or workers (15). This is probably the reason for the differences between the MW and CW bacterioplankton in the seedling stage based on unweighted Unifrac distance. In addition, the epilithic biofilm of cultivated macroalgae could be another factor which contributed to the rare species in the water column (33). However, these foreign or attached bacteria were often not abundant enough to affect the overall abundance composition of bacterioplankton (36). At the mature stage, long-term seaweed cultivation will cause the selective enrichment of some specific species, thereby changing the relative abundance of some bacteria (33). This is the possible reason for significant differences in the bacterioplankton abundance compositions between the MW and CW samples at the mature stage. More importantly, although the bacterioplankton abundance composition changed after the kelp culture, it maintained the biodiversity stability of bacterioplankton in the culture area.

**FIG 3:**
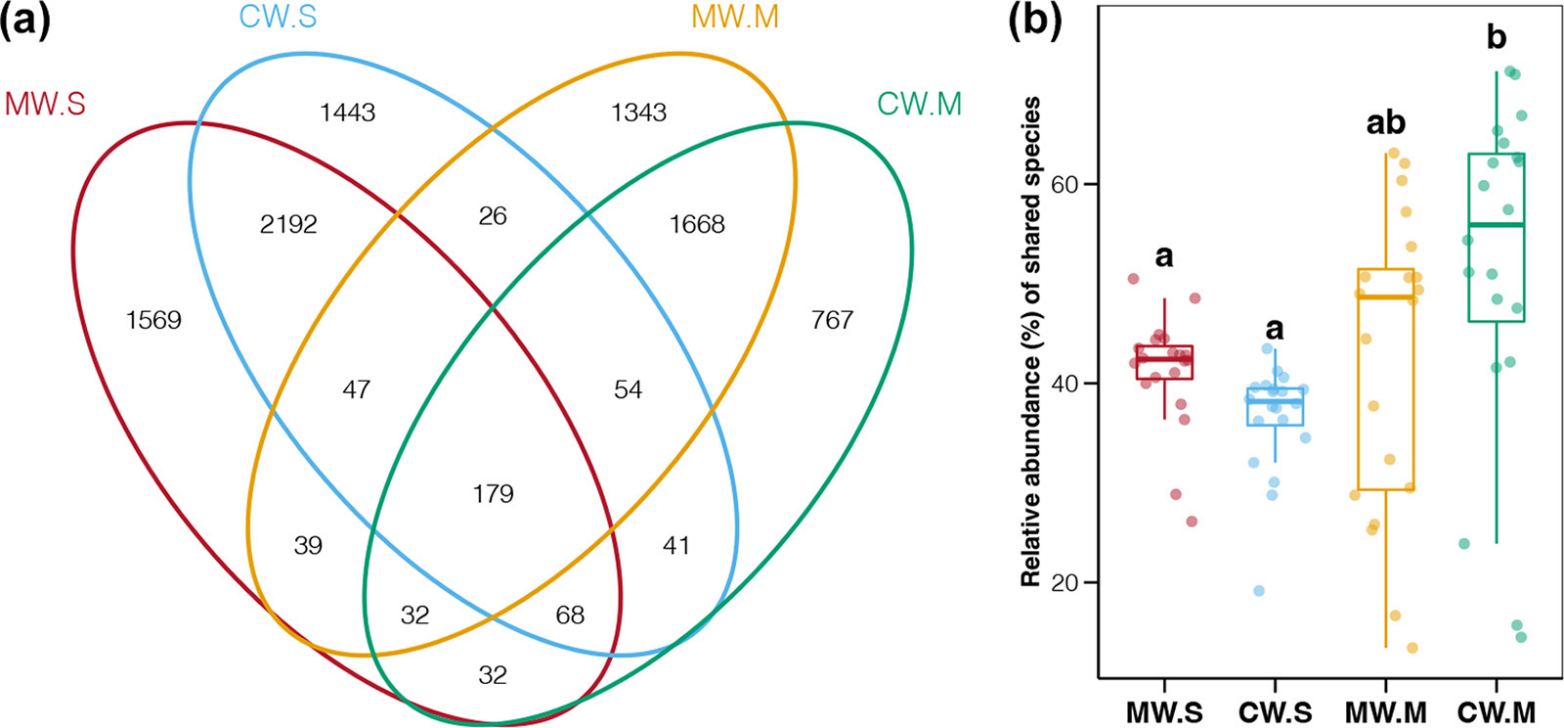
(a) Shared and unique amplicon sequence variants (ASVs) among different bacterioplankton communities. (b) Differences in total relative abundances of shared ASVs among different bacterioplankton communities. Different lowercase letters above each box in the same panel represent significant differences between groups (Tukey’s HSD test, *P < *0.05).

According to Venn diagram analysis, 179 ASVs were shared among all four sample groups; in contrast, the numbers of unique ASVs in each group were obviously higher (Fig. 3a). These results hinted that the bacterioplankton biodiversity in the study samples was mainly contributed by non-core bacteria, consistent with being obtained from diverse habitats at different scales (37, 38). In general, the number of core taxa in microbial communities from a specific habitat is limited but occupies dominant relative abundances (39). In this study, the average total relative abundances of these shared ASVs were 40% to 60% in different groups (Fig. 3a). This reflected the fact that changes in bacterioplankton abundance composition were mainly governed by a few core species; in contrast, variations in biodiversity were primarily contributed by the larger numbers of low-abundance uncommon species (40). Moreover, an increase in the relative abundances of shared ASVs was found in samples at the mature stage compared to those at the seedling stage (Fig. 3b). Notably, the increase in the total relative abundance of shared ASVs in MW samples was lower than that in CW samples (Fig. 3b). Differences in the number of unique ASVs in MW samples from seedling to mature stages (1,569 to 1,343) were also lower than those in CW samples (1,443 to 767; Fig. 3a). These results supported the speculation from the beta diversity analysis based on unweighted and weighted Unifrac distances described above (Fig. 2). Beta diversity and core taxa analyses showed that kelp cultivation favored the survival of uncommon bacteria and thus maintained the biodiversity of bacterioplankton communities.

### Kelp culture enhances biogeochemical cycling capacities.

**FIG 4:**
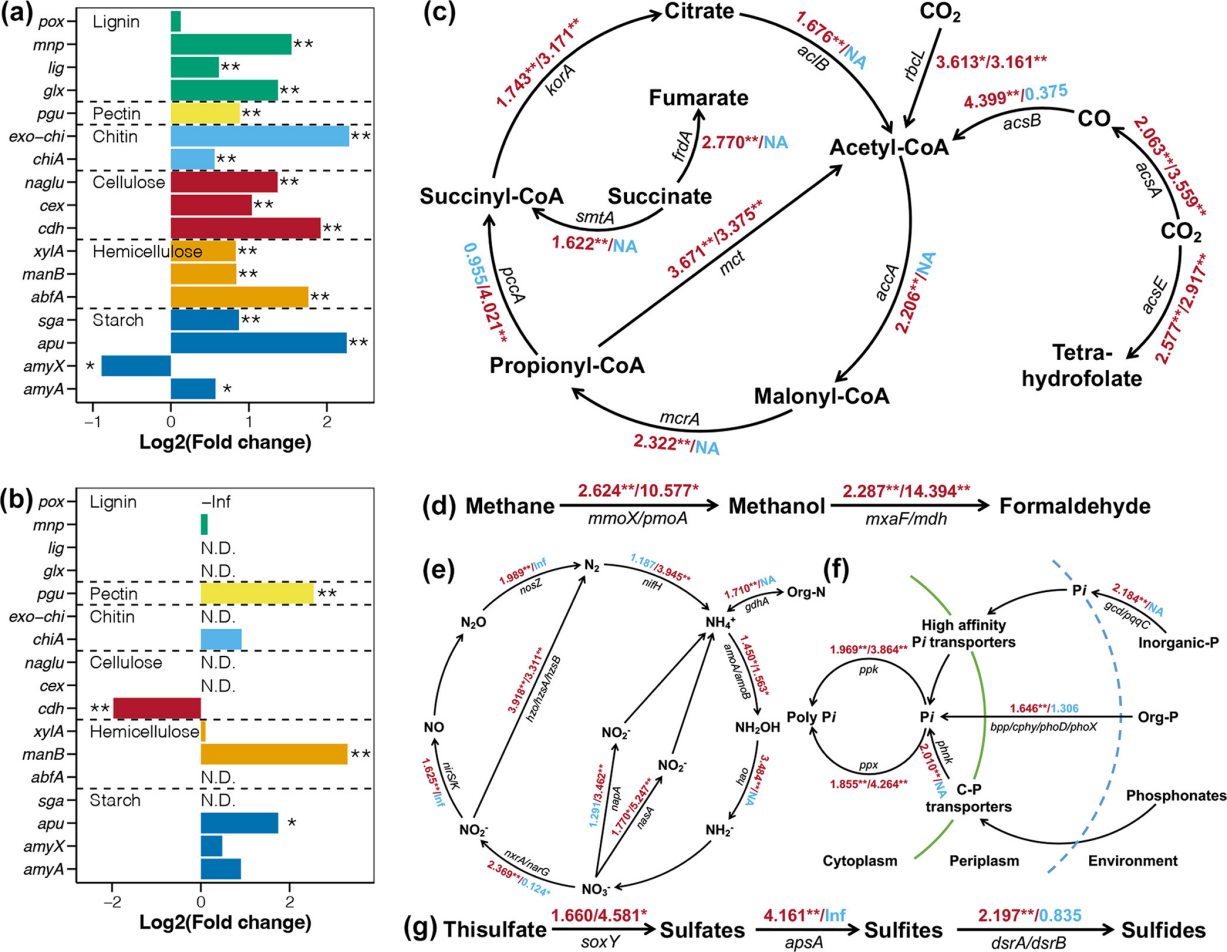
Fold changes in relative abundances of different genes between MW and CW samples at the seedling and mature stages. (a) C degradation genes at the seedling stage. (b) C degradation genes at the mature stage. (c) C fixation genes. (d) Methane metabolism genes. (e) N cycling genes. (f) P cycling genes. (g) S cycling genes. Values before and after “/” represent fold changes in relative abundances of each gene between MW and CW samples at the seedling and mature stages, respectively. Student’s *t* test for significant test, **, *P < *0.01; *, *P < *0.05. ND and NA represent no detection of this gene in either MW or CW samples from the same stage.

The relative abundances of microbial genes related to biogeochemical cycles in studied bacterioplankton communities were measured by a HT-qPCR chip, and the comparisons between MW and CW samples at both the seedling and mature stages are shown in Fig. 4. Compared to those in CW samples, the relative abundances of most biogeochemical cycling genes were significantly increased in MW samples, regardless of kelp life stage (Student’s *t* test, *P < *0.05). Moreover, the fold changes of these increased genes were higher in the mature stage than in the seedling stage. In addition, some biogeochemical cycling functions, including cellulose degradation (*cdh*) (Fig. 4b), CO utilization (*acsB*) (Fig. 4c), and nitrate dissimilatory reduction (*nxrA*/*narG*) (Fig. 4e), were found to be inhibited in kelp culture areas at the mature stage. Furthermore, kelp culture activities did not significantly affect organic phosphate utilization (Fig. 4f) or sulfide synthesis (Fig. 4g) in bacterioplankton at the mature stage.

Cultured seaweed is considered an important blue carbon sink (7) which is estimated to absorb 32,000 tons of carbon each year along the coasts of Japan (41). In addition, large-scale seaweed cultivation is also believed to be a solution for coastal eutrophication (42). Lower nutrient contents have been observed in seaweed cultivation zones compared to non-mariculture zones (14, 15). Moreover, polyculture of other species with seaweed has been proven to remove excess nitrogen and phosphate (43). However, most related investigations have only considered the direct absorption of these substances by the seaweed itself (44–46). Admittedly, direct absorption by seaweed is the dominant element fixation pathway; however, the microbes related to seaweed cultivation in biogeochemical cycles also contribute substantially (47). Our study results revealed that seaweed culture activities effectively promoted the biogeochemical cycles of bacterioplankton in the culture areas. These processes could include direct fixation of nutrients by microorganisms as well as further utilization by other organisms (e.g., seaweed) after microbial transformation (48).

Notably, some genes were undetected in the mature stage, which could be due to the lower temperature and bacterioplankton biomass during this stage (Fig. S3 and S4). Low biomass means that less microbial DNA is obtained for the same amount of water, which may result in the abundance of many genes below the detection limit of HT-qPCR. In this study, we only compared differences in the relative abundances of biogeochemical cycling genes to avoid biomass effects due to seasonal variation, which would represent the latent ability of microbes and not the absolute values. The lower bacterioplankton biomass during the mature stage implied that absolute abundance of these undetected genes was low even if they were present. The phenomenon where genes in the mature stage were not detected was particularly obvious in the C-degradation genes (Fig. 4a and b). These results might indirectly reflect the relatively low capacity for C utilization in this season.

10.1128/msystems.00002-23.4 FIG S4 Differences in bacterial biomass between seedling and mature stages. Download FIG S4, PDF file, 0.02 MB. Copyright © 2023 Sun et al. 2023 Sun et al. https://creativecommons.org/licenses/by/4.0/ This content is distributed under the terms of the Creative Commons Attribution 4.0 International license.

### Relationships between the co-occurrence modules of bacterioplankton and biogeochemical cycles.

**FIG 5:**
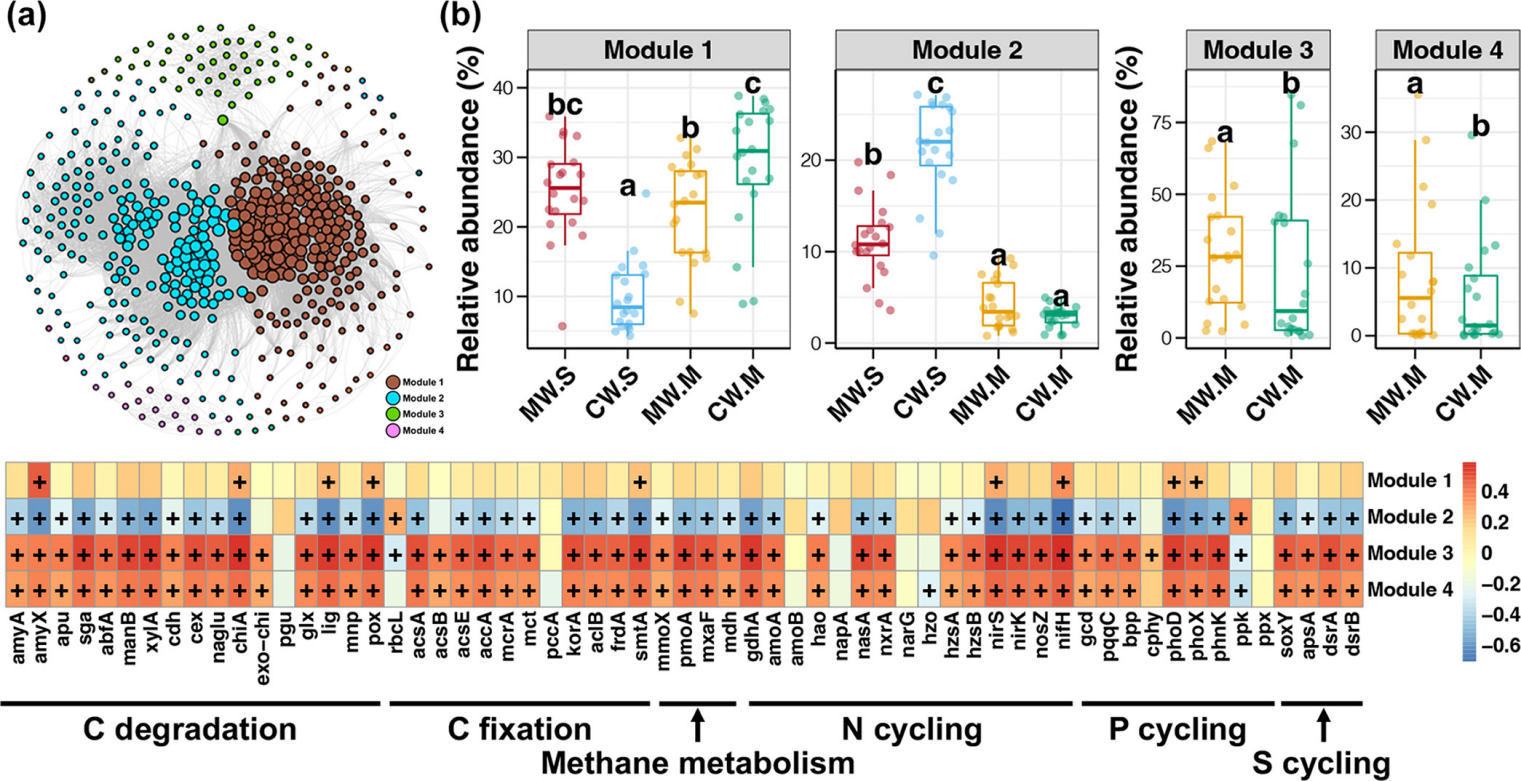
(a) Co-occurrence network of bacterioplankton in MW and CW samples at both the seedling and mature stages. (b) Differences in relative abundances of dominant co-occurrence modules among different bacterioplankton communities. Different lowercase letters above each box in the same panel represent significant differences between groups (Tukey’s HSD test, *P* < 0.05). (c) Heatmap showing correlations between co-occurrence modules and biogeochemical cycling genes. A plus sign (+) indicates significant correlation (*P < *0.05).

Rather than simple accumulation of individual populations in macroorganisms, microorganisms in natural ecosystems form complex ecological networks which are critical to maintain ecosystem functions (49). We explored the co-occurrence patterns among bacterioplankton in MW and CW samples at both the seedling and mature stages. Our results showed that the co-occurrence network had obvious modularity with four dominant ASV modules (Fig. 5a). Among these, modules 1 and 2 were present in water from both seedling and mature stages, while modules 3 and 4 almost only existed in samples from the mature stage (Fig. 5b). The relative abundance of module 1 was unchanged in MW samples between the seedling and mature stages; however, it was significantly lower in CW samples from the seedling stage and higher in those from the mature stage (Tukey’s HSD test, *P < *0.05). The relative abundance of module 2 was significantly higher in water from the seedling stage compared to water from the mature stage (*P < *0.05). Moreover, kelp culture significantly decreased the relative abundance of module 2 in the seedling stage (*P < *0.05). The relative abundances of modules 3 and 4 were significantly higher in WM samples compared to CW samples in the mature stage (*P < *0.05).

Based on the taxa of nodes in each module, most members of module 2 belonged to shared ASVs among all different groups (Fig. 3a), while the nodes in modules 1, 3, and 4 were occupied by unique ASVs (Fig. S5). These results indicated the bacterioplankton biodiversity in kelp cultivation areas regulated by uncommon bacterial species could influence their co-occurrence patterns. Furthermore, a correlation analysis revealed that most of the relative abundance of biogeochemical cycling genes were negatively correlated with the relative abundance of module 2, but positively correlated with the relative abundances of modules 3 and 4 (Spearman’s correlation, *P < *0.05, Fig. 5c). This information implied that the capacities of biogeochemical cycles in kelp cultivation areas might correlate with the co-occurrence patterns of bacterioplankton regulation by biodiversity variation. Rare taxa (low-abundance uncommon species) maintain bacterioplankton biodiversity in kelp cultivation areas, increasing the relative abundances of some modules composed of them and decreasing the module containing core bacteria. In addition, microbial communities with rich diversity and rarer taxa have been reported to have more ecological stability at both the community and functional levels (50, 51). The important roles of rare taxa in maintaining the stability of microbial communities and ecosystem functions have been increasingly demonstrated in diverse terrestrial ecosystems (37, 52, 53). We uncovered the potential roles of low-abundance uncommon bacteria in maintaining bacterioplankton biodiversity and affecting the biogeochemical cycles in coastal ecosystems with kelp cultivation.

10.1128/msystems.00002-23.5 FIG S5 Ratio of core and non-core amplicon sequence variants (ASVs) in different modules. Download FIG S5, PDF file, 0.00 MB. Copyright © 2023 Sun et al. 2023 Sun et al. https://creativecommons.org/licenses/by/4.0/ This content is distributed under the terms of the Creative Commons Attribution 4.0 International license.

### Effects of kelp cultivation on B-EF relationships.

**FIG 6:**
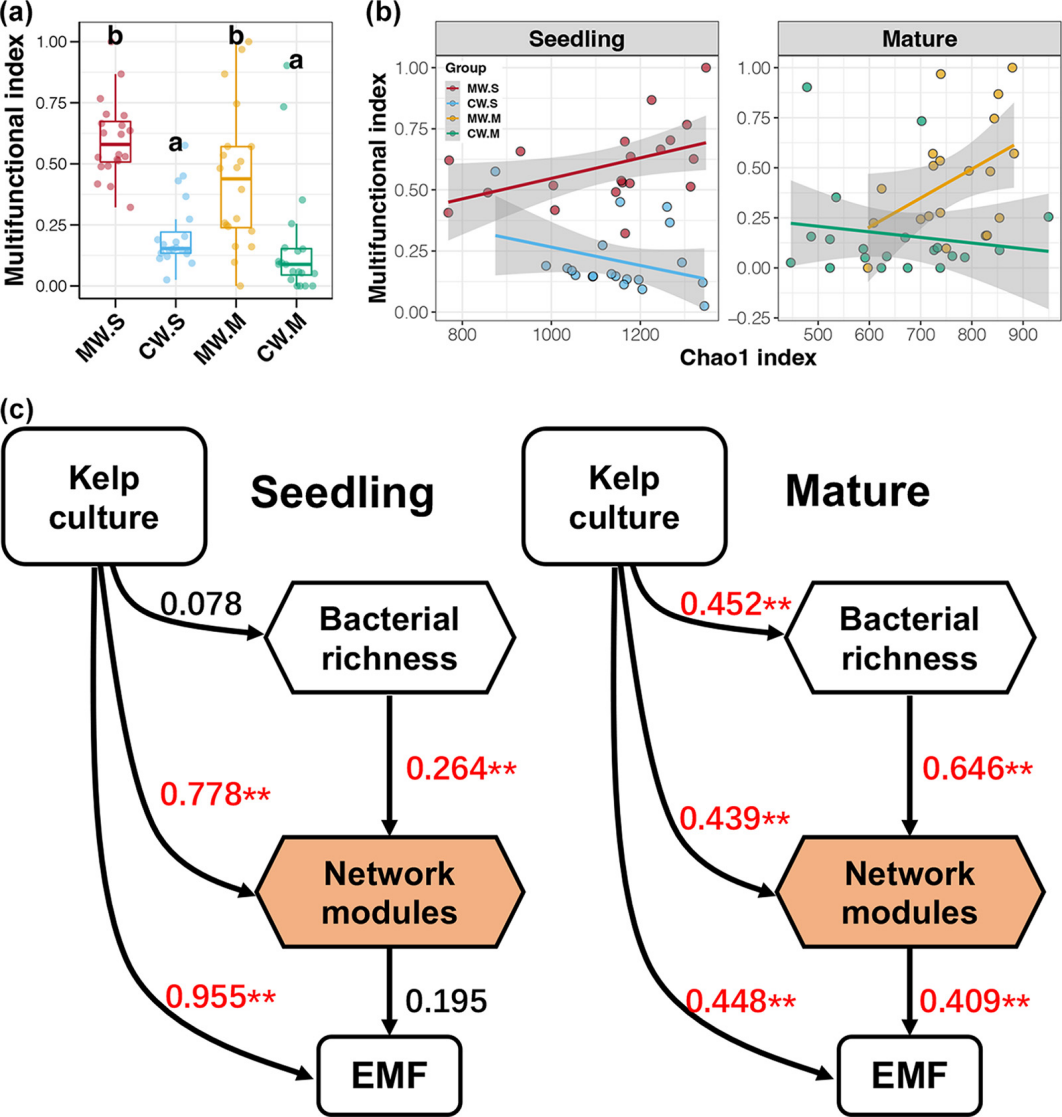
(a) Differences in multifunctional index among different samples. Different lowercase letters above each box in the same panel represent significant differences between groups (Tukey’s HSD test, *P < *0.05). (b) Linear regression between the Chao1 index and multifunctional index of bacterioplankton from different water samples. (c) Partial least-squares path modeling showing the effects of kelp culture on ecosystem biogeochemical cycling via bacterioplankton richness and co-occurrence modules. EMF, ecosystem multifunctionality.

In general, an ecosystem can provide multiple functions and services, including provisioning (production and quality), support (biogeochemical cycles and stability), regulation (carbon fixation and pathogen control), and cultural services (natural scenery and tourist industry) (54). To deeply evaluate the effects of kelp cultivation on B-EF relationships, we first calculated a multifunctional index based on the relative abundance of measured biogeochemical cycling genes. The results showed that the multifunctional index of bacterioplankton in the kelp culture area was significantly higher than that in non-culture control area, regardless of kelp life stage (Tukey’s HSD test, *P < *0.05, Fig. 6a). This suggested that kelp cultivation enhanced the ecosystem functions related to biogeochemical cycles in coastal ecosystems, which was consistent with expectations (55).

Biodiversity has multiple aspects, including taxonomic, phylogenetic, and functional attributes, and different biodiversity indices measure different organism features (20). Different biodiversity aspects do not correlate with each other and may have different effects on ecosystem functions (56). In this study, we performed correlations between the four alpha diversity indices of bacterioplankton and multifunctional index to assess the B-EF relationships associated with kelp cultivation. Only the Chao1 index had significant correlations with the multifunctional index in MW samples from both the seedling and mature stages (linear regression, *P < *0.05, Fig. 6b and Table S3), indicating the potential relationship between bacterioplankton richness and the biogeochemical cycles in kelp cultivation areas. As the most fundamental aspect of biodiversity, the relationship between species richness and ecosystem functions has been demonstrated by many experimental and monitoring studies (57–59). However, we present the first evidence for the relationship between bacterial richness and biogeochemical cycles in kelp cultivation areas.

10.1128/msystems.00002-23.8 TABLE S3 Linear regression between alpha diversity indices of bacterioplankton and multifunctional index in different groups. Download Table S3, DOCX file, 0.01 MB. Copyright © 2023 Sun et al. 2023 Sun et al. https://creativecommons.org/licenses/by/4.0/ This content is distributed under the terms of the Creative Commons Attribution 4.0 International license.

Stressors can affect B-EF relationships in different ways, including indirect effects on ecosystem functions via biodiversity, effects on B-EF relationships, and direct effects on ecosystem functions (19). To further explore the direct and indirect effects of kelp culture on ecosystem functions, partial least-squares path modeling (PLS-PM) was conducted for the seedling and mature stages, respectively (Fig. 6c). In the seedling stage, the effects of kelp culture on ecosystem multifunctionality were mainly direct, followed by indirect effects through bacterioplankton modules. In the mature stage, the contributions of direct effects of kelp culture on ecosystem multifunctionality declined, and indirect effects mediated by bacterioplankton richness increased. Integrating network results into the B-EF relationship is considered to provide a better mechanistic understanding of how biodiversity relates to ecosystem functions (60). These results revealed the effects of kelp cultivation on the bacterial richness and B-EF relationship by regulating co-occurrence modules to indirectly influence the biogeochemical cycles in coastal ecosystems.

### Conclusions.

In this study, we profiled the bacterioplankton communities in a representative kelp cultivation area and adjacent non-culture coasts in both the seedling and mature stages. Our findings revealed that kelp cultivation activities maintained bacterioplankton biodiversity in the culture area under natural seasonal change. The higher biodiversity of bacterioplankton in kelp cultivation areas is contributed by low-abundance non-common bacteria, which were not present in all culture areas during the culture cycle. Moreover, the relative abundances of genes involving biogeochemical cycles were measured using a HT-qPCR chip. The results showed a more powerful ability of biogeochemical cycles in kelp cultivation areas compared to that in non-culture coasts. The higher bacterioplankton biodiversity in kelp cultivation areas regulated microbial interactions to indirectly influence biogeochemical cycles, and thus enhanced the ecosystem functions of coasts with kelp cultivation. The findings of this study allow us to better understand the effects of kelp cultivation on the biodiversity and biogeochemical cycles of bacterioplankton in coastal ecosystems and reveal the mechanisms by which kelp cultivation influences B-EF relationships.

## MATERIALS AND METHODS

### Seaweed cultivation area.

Lvshunkou District (Dalian, Liaoning Province, China) is located at the southernmost tip of the Liaodong Peninsula. Suitable temperature, sufficient sunlight, and smooth water exchange provide excellent conditions for seaweed cultivation. The seaweed cultivation area on the coast of Lvshunkou District exceeds 1,700 ha., with kelp (Laminaria japonica) as the main species. The kelp seedlings are placed into the culture areas in November each year, and the mature kelp is harvested in May the following year. As the Lvshunkou District is one of the most important kelp-producing areas in China, “Lvshun Kelp” has been certified as a national geographical indication product. The annual kelp output in Lvshunkou District can reach 300,000 tons and generates nearly one billion Chinese yuan in output value.

### Sample collection and DNA extraction.

Surface water samples were collected from the kelp culture area and adjacent non-culture area in December 2021 (seedling stage) and May 2022 (mature stage), respectively. Twenty surface water samples (~2 L) were collected at random locations (30- to 50-m apart from each other) in each area at each stage. A total of 80 samples were obtained and named using the convention MW/CW.S/M.N, in which MW/CW indicated a mariculture (MW) or control water (CW) sample, S/M represented the sampling stage (S for seedling and M for mature), and N represented the serial number of the sampling order (1 to 20 for each area in each stage). All samples were immediately placed in an ice box and transported to the laboratory within 8 h of collection.

After arriving at the lab, water samples were immediately filtered by a water filtration apparatus with 0.22-μm Durapore membrane filters (Millipore, MA) to concentrate the bacterial cells. Bacterial DNA was extracted from each filter membrane using a PowerWater DNA isolation kit (Mo Bio Laboratories, CA) following the manufacturer’s instructions. Agarose gel electrophoresis (1.5% concentration) was used to detect successful DNA extraction. The concentration and purity of successfully extracted DNA were measured by a NanoPhotometer Classic Launched (Implen, Munich, Germany). All DNA samples were stored at −20°C for further application.

### 16S rRNA sequencing.

The bacterial 16S rRNA V3 to V4 hypervariable region of each sample was amplified by PCR with primers 341F to 806R combined with Illumina adapter sequences, a pad, a linker of two bases, and barcodes on the reverse primer (61). The PCR products were then sequenced on the Illumina NovaSeq 6000 platform with the PE250 strategy at Biozeron Biotech. Co., Ltd. (Shanghai, China). Raw paired-end reads were assigned to each sample based on their unique barcodes. Next, a standard pipeline using the DADA2 plug-in unit in QIIME2 was performed for quality control and clustering to obtain the amplicon sequence variants (62). Each ASV was assigned a taxonomy based on the SILVA database (release 138) (63). Non-bacterial ASVs (i.e., chloroplast and archaea) and singletons (the number of a specific ASV was one) were abandoned. Finally, the ASV abundance tables were normalized using a standard number of reads according to the sample with the lowest read number (25,796).

### Measurement of nutrient cycling genes.

A qPCR-based chip, QMEC, containing 71 primers for microbial functional genes in C, N, P, and S biogeochemical cycling was used to measure the biogeochemical cycling capacity of each sample (64). DNA of each sample were used as the template for QMEC detection quantified by HT-qPCR (SmartChip Real-Time PCR system, WaferGen Biosystems, Fremont, CA) using the bacterial 16S rRNA gene (F525/R907) as the reference gene (65). qPCR was performed according to the standard method described by Zheng et al. (64) with three replicates for each sample. Results with multiple melting peaks or amplification efficiencies of <80% or >120% were excluded by the SmartChip qPCR software. Results with a threshold cycle (*C_T_*) of <31 were used for further analysis. Relative gene abundance was defined as the proportion of the abundance of a functional gene to the abundance of the 16S rRNA gene, which was calculated as described by Looft et al. (66).

### Statistical analysis.

All statistical analysis were executed in R v4.0.2 and visualized by the “ggplot2” package or Microsoft PowerPoint. Four alpha diversity indices of bacterioplankton communities, including Chao1, Shannon, Peilou, and phylogenetic diversity, were calculated using the “vegan” package. Differences in alpha diversity indices among different samples were compared by ANOVA with Tukey’s HSD test. Weighted and unweighted distances between bacterioplankton were obtained by the “GuniFrac” package. PCoA and PERMANOVA based on the beta diversity distances were accomplished using the “vegan” package. A Venn diagram analysis was performed using the “VennDiagram” package to identify the shared bacteria ASVs among different samples. Differences in the total relative abundance of shared ASVs among different samples were also assessed by ANOVA with Tukey’s HSD test.

The average fold change of relative abundance of nutrient cycling genes between the MW and CW samples in each culture stage was calculated, and differences were evaluated by Student’s *t* test. The co-occurrence pattern of bacterioplankton was constructed based on Spearman rank correlations among all ASVs detected in all samples. Co-occurrence events were identified with statistically robust correlations (|correlation coefficient| > 0.8 with *P* < 0.05) (67). *P* values were adjusted using the Benjamini-Hochberg method (68). Bacterial modules were recognized from the co-occurrence network using the “igraph” package, and the total relative abundances of different modules among different samples were compared by ANOVA with Tukey’s HSD test. Correlations between the relative abundances of bacterial modules and nutrient cycling genes were analyzed using Spearman rank correlation.

Multifunctionality is an important ecological and management concept and provides the basis for a solid statistical approach that enables the synthesis of the many diverse functions (69). According to the method used for soil multifunctional index, a multifunctional index for studied samples was calculated based on the relative abundance of biogeochemical cycling genes (70). Briefly, the biogeochemical cycling genes were classified into six variables representative of C degradation, C fixation, methane metabolism, N cycling, S cycling, and P cycling according to their functions. Next, a principal coordinate analysis score for each of the six latent variables making up the composite variable was calculated. The score of PCs for each latent variable was then averaged after weighting by the proportion of variation each explained by each eigenvalue, and then scaled between 0 and 1. The overall multifunctional index was computed by averaging the scaled scores of six latent variables. Differences in the multifunctional index among different samples were estimated by ANOVA with Tukey’s HSD test. Relationships of multifunctional index with the alpha diversity and bacterial modules in bacterioplankton were evaluated by linear regression. Finally, PLS-PM was performed using the “PLSPM” package to quantify the effects of kelp culture on bacterioplankton and the relationships between biodiversity and ecosystem functions.

### Data availability.

16S rRNA amplicon sequencing data have been deposited in the National Center for Biotechnology Information SRA database under BioProject no. PRJNA915467.
