# Supplementary figures and images for: Kelp Culture Enhances Coastal Biogeochemical Cycles by Maintaining Bacterioplankton Richness and Regulating Its Interactions
Source: mSystems. 2023 Feb 16;8(2):e00002-23. doi: 10.1128/msystems.00002-23 (PMC10134829; doi:10.1128/msystems.00002-23)

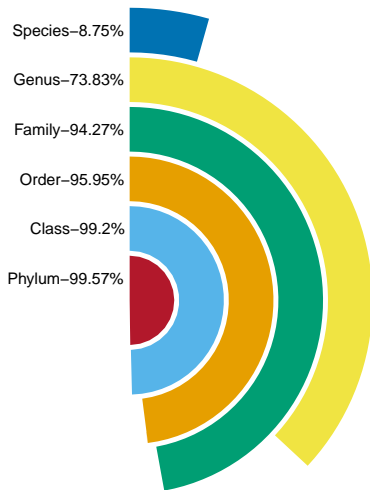

Supplement: FIG S1 [file msystems.00002-23-s0001.pdf]

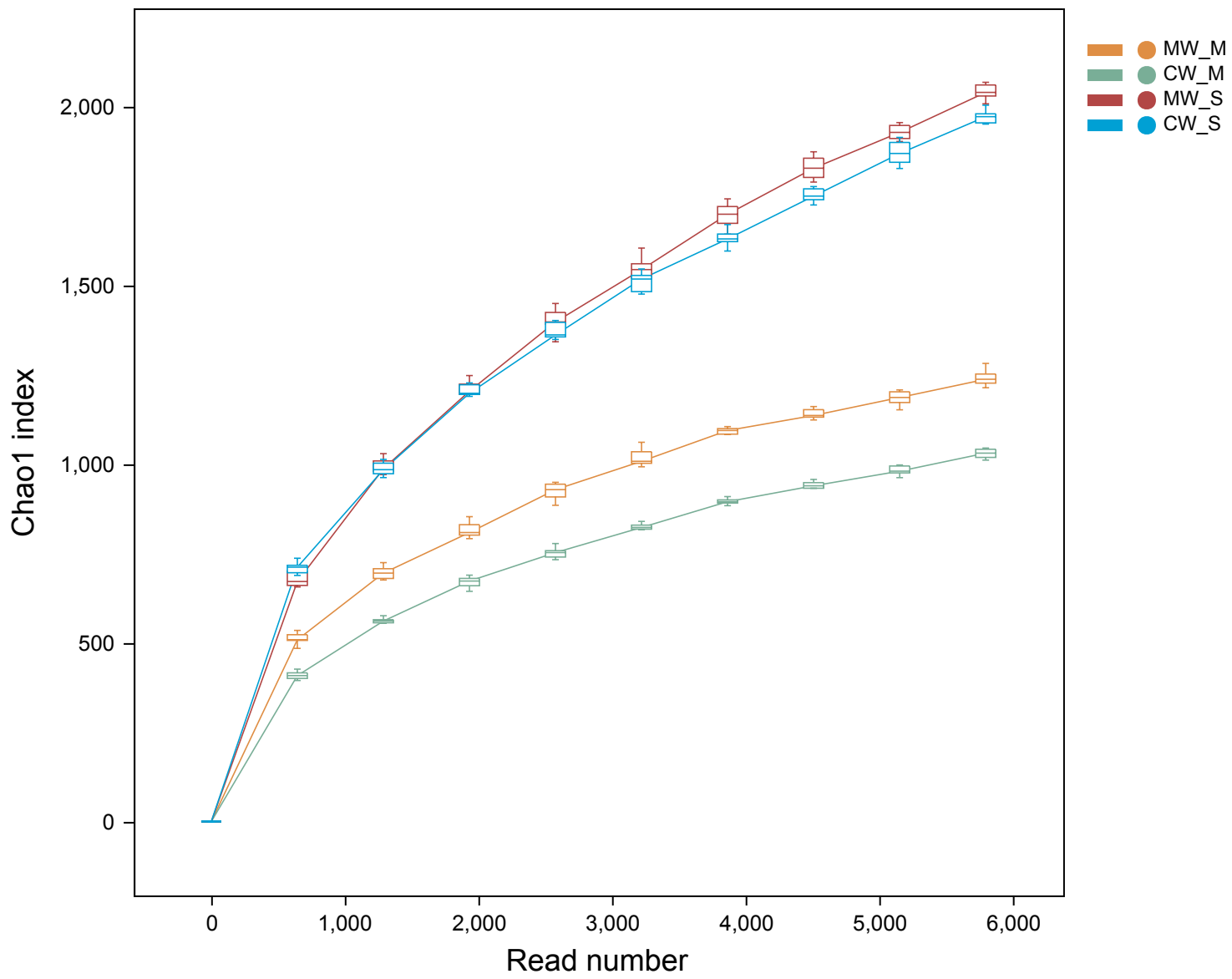

Supplement: FIG S2 [file msystems.00002-23-s0002.pdf]

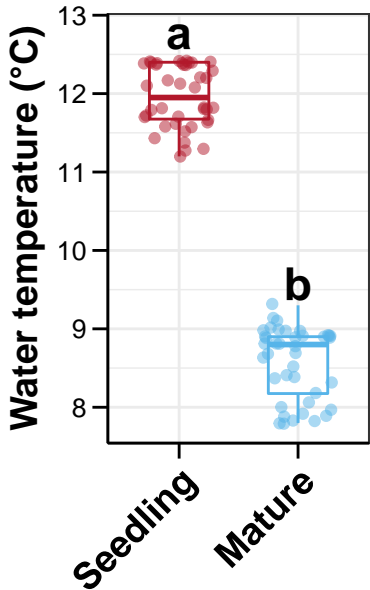

Supplement: FIG S3 [file msystems.00002-23-s0003.pdf]

Bacterial biomass

( $10^9$  16S copies per L water)

2.0  
1.5  
1.0  
0.5  
0.0

**a**

Seedling

**b**

Mature

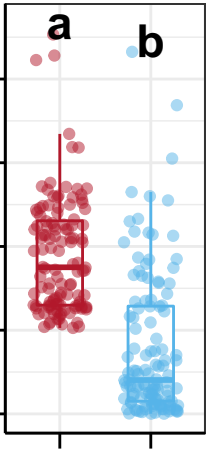

Supplement: FIG S4 [file msystems.00002-23-s0004.pdf]

Type

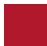

Core

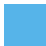

Non-core

Ratio of ASVs

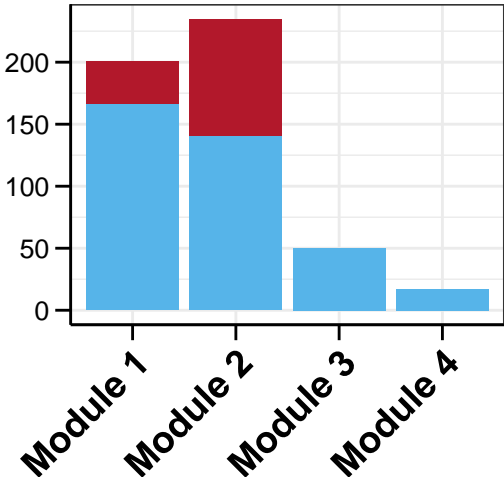

Supplement: FIG S5 [file msystems.00002-23-s0005.pdf]
