# Supplementary material for: Kelp Culture Enhances Coastal Biogeochemical Cycles by Maintaining Bacterioplankton Richness and Regulating Its Interactions
Source: mSystems. 2023 Feb 16;8(2):e00002-23. doi: 10.1128/msystems.00002-23 (PMC10134829; doi:10.1128/msystems.00002-23)
Supplement: TABLE S1 [file msystems.00002-23-s0006.docx]

**Table S1.** Statistics of sequencing data.

| **Sample ID** | **Input** | **Filtered** | **Denoised** | **Merged** | **Non-chimeric** | **Non-singleton** |
| --- | --- | --- | --- | --- | --- | --- |
| MW.M.1 | 144,111 | 138,143 | 135,790 | 131,858 | 113,272 | 112,745 |
| MW.M.2 | 136,727 | 132,439 | 129,757 | 125,201 | 102,304 | 101,696 |
| MW.M.3 | 125,213 | 120,770 | 118,182 | 113,516 | 95,309 | 94,660 |
| MW.M.4 | 118,679 | 112,532 | 109,684 | 103,641 | 84,178 | 83,517 |
| MW.M.5 | 113,462 | 108,708 | 105,717 | 99,451 | 81,847 | 81,104 |
| MW.M.6 | 94,873 | 90,749 | 88,939 | 85,201 | 64,603 | 64,016 |
| MW.M.7 | 128,398 | 123,701 | 121,134 | 115,479 | 99,999 | 99,478 |
| MW.M.8 | 128,244 | 121,711 | 119,425 | 115,078 | 99,882 | 99,352 |
| MW.M.9 | 141,789 | 135,795 | 132,940 | 125,902 | 103,045 | 102,430 |
| MW.M.10 | 132,813 | 127,461 | 124,686 | 117,605 | 97,113 | 96,388 |
| MW.M.11 | 129,113 | 124,634 | 122,432 | 118,838 | 102,487 | 102,101 |
| MW.M.12 | 139,252 | 131,816 | 129,759 | 126,153 | 109,588 | 109,212 |
| MW.M.13 | 106,839 | 101,758 | 97,998 | 91,143 | 67,099 | 66,007 |
| MW.M.14 | 149,220 | 143,417 | 141,124 | 135,450 | 116,772 | 116,211 |
| MW.M.15 | 131,802 | 127,249 | 125,026 | 121,594 | 104,157 | 103,626 |
| MW.M.16 | 130,206 | 124,560 | 123,078 | 120,613 | 111,788 | 111,497 |
| MW.M.17 | 133,180 | 127,364 | 125,341 | 122,358 | 101,629 | 101,178 |
| MW.M.18 | 134,022 | 126,951 | 124,424 | 120,277 | 99,077 | 98,457 |
| MW.M.19 | 134,231 | 121,560 | 120,354 | 118,621 | 109,815 | 109,638 |
| MW.M.20 | 131,212 | 123,027 | 121,647 | 119,607 | 104,343 | 104,104 |
| CW.M.1 | 112,028 | 107,130 | 104,007 | 97,966 | 71,850 | 71,073 |
| CW.M.2 | 132,838 | 127,327 | 125,542 | 122,359 | 108,001 | 107,667 |
| CW.M.3 | 112,181 | 107,933 | 106,167 | 103,417 | 80,865 | 80,521 |
| CW.M.4 | 97,917 | 93,245 | 89,805 | 83,997 | 51,742 | 50,740 |
| CW.M.5 | 137,961 | 133,293 | 131,409 | 128,501 | 105,589 | 105,201 |
| CW.M.6 | 132,090 | 127,124 | 125,184 | 121,954 | 105,149 | 104,787 |
| CW.M.7 | 117,016 | 112,073 | 110,347 | 106,351 | 95,652 | 95,392 |
| CW.M.8 | 106,079 | 101,005 | 98,102 | 91,634 | 68,898 | 68,239 |
| CW.M.9 | 106,885 | 99,738 | 96,924 | 91,293 | 71,645 | 70,684 |
| CW.M.10 | 101,970 | 97,700 | 95,651 | 91,849 | 73,017 | 72,536 |
| CW.M.11 | 113,804 | 109,514 | 107,865 | 105,237 | 81,194 | 80,824 |
| CW.M.12 | 117,212 | 111,818 | 111,021 | 109,691 | 99,027 | 98,905 |
| CW.M.13 | 125,997 | 121,526 | 120,025 | 117,838 | 105,013 | 104,796 |
| CW.M.14 | 123,167 | 119,024 | 117,823 | 115,949 | 99,457 | 99,290 |
| CW.M.15 | 122,676 | 118,306 | 117,306 | 115,805 | 106,291 | 106,137 |
| CW.M.16 | 124,965 | 119,048 | 117,304 | 114,320 | 93,897 | 93,524 |
| CW.M.17 | 135,166 | 129,343 | 126,634 | 120,253 | 95,627 | 94,734 |
| CW.M.18 | 147,775 | 142,003 | 140,447 | 137,944 | 128,997 | 128,759 |
| CW.M.19 | 174,128 | 167,380 | 165,824 | 162,976 | 150,432 | 150,193 |
| CW.M.20 | 112,872 | 107,186 | 105,795 | 103,798 | 90,163 | 89,940 |
| MW.S.1 | 70,910 | 64,741 | 62,021 | 48,965 | 27,603 | 25,796 |
| MW.S.2 | 122,745 | 116,122 | 112,755 | 106,587 | 75,600 | 74,800 |
| MW.S.3 | 120,025 | 113,941 | 110,518 | 104,573 | 73,629 | 72,767 |
| MW.S.4 | 114,923 | 107,566 | 104,205 | 97,603 | 69,987 | 69,155 |
| MW.S.5 | 151,806 | 143,222 | 137,987 | 126,169 | 99,049 | 97,320 |
| MW.S.6 | 134,435 | 126,512 | 122,379 | 114,537 | 84,679 | 83,625 |
| MW.S.7 | 103,316 | 97,436 | 93,117 | 83,486 | 56,499 | 55,196 |
| MW.S.8 | 129,647 | 121,413 | 116,583 | 107,349 | 69,203 | 67,626 |
| MW.S.9 | 118,958 | 112,649 | 108,167 | 98,044 | 61,670 | 59,847 |
| MW.S.10 | 121,898 | 115,473 | 110,785 | 100,546 | 66,090 | 64,315 |
| MW.S.11 | 167,919 | 159,700 | 156,770 | 151,776 | 132,266 | 131,713 |
| MW.S.12 | 83,255 | 75,821 | 71,624 | 56,775 | 35,026 | 32,876 |
| MW.S.13 | 77,904 | 72,327 | 68,404 | 53,119 | 32,917 | 31,220 |
| MW.S.14 | 101,491 | 96,522 | 92,931 | 85,411 | 62,045 | 60,873 |
| MW.S.15 | 104,830 | 99,290 | 95,288 | 86,919 | 59,957 | 58,475 |
| MW.S.16 | 104,829 | 97,183 | 93,063 | 85,203 | 70,606 | 69,389 |
| MW.S.17 | 107,683 | 102,392 | 98,611 | 89,996 | 62,360 | 61,105 |
| MW.S.18 | 107,810 | 102,536 | 99,396 | 92,697 | 74,150 | 73,427 |
| MW.S.19 | 108,884 | 102,981 | 98,657 | 88,530 | 62,467 | 60,856 |
| MW.S.20 | 113,393 | 105,902 | 101,771 | 92,714 | 72,401 | 71,260 |
| CW.S.1 | 108,352 | 103,006 | 97,758 | 86,414 | 66,079 | 64,232 |
| CW.S.2 | 99,712 | 94,342 | 90,429 | 81,713 | 67,923 | 66,889 |
| CW.S.3 | 98,443 | 93,278 | 88,350 | 77,630 | 60,695 | 59,372 |
| CW.S.4 | 143,120 | 133,107 | 126,335 | 111,556 | 82,370 | 80,230 |
| CW.S.5 | 111,108 | 106,483 | 101,280 | 89,329 | 70,941 | 69,154 |
| CW.S.6 | 104,613 | 98,542 | 95,153 | 87,955 | 76,960 | 76,250 |
| CW.S.7 | 102,244 | 96,756 | 93,519 | 87,122 | 65,705 | 64,594 |
| CW.S.8 | 123,450 | 116,488 | 112,760 | 105,022 | 93,783 | 92,856 |
| CW.S.9 | 131,229 | 125,153 | 119,548 | 106,920 | 83,168 | 81,292 |
| CW.S.10 | 109,665 | 103,908 | 99,827 | 90,714 | 77,300 | 76,424 |
| CW.S.11 | 110,009 | 104,061 | 99,816 | 91,022 | 73,103 | 71,930 |
| CW.S.12 | 97,842 | 91,379 | 87,667 | 79,686 | 56,794 | 55,614 |
| CW.S.13 | 117,078 | 111,140 | 105,932 | 95,209 | 79,039 | 77,622 |
| CW.S.14 | 84,809 | 80,148 | 76,437 | 68,270 | 54,411 | 53,536 |
| CW.S.15 | 106,654 | 100,598 | 96,153 | 86,466 | 74,018 | 72,951 |
| CW.S.16 | 84,599 | 76,141 | 71,503 | 49,139 | 37,657 | 36,330 |
| CW.S.17 | 132,895 | 125,764 | 120,017 | 107,741 | 90,351 | 88,705 |
| CW.S.18 | 164,055 | 154,141 | 147,894 | 133,850 | 112,963 | 111,450 |
| CW.S.19 | 110,413 | 103,388 | 99,532 | 90,722 | 76,812 | 75,904 |
| CW.S.20 | 128,133 | 120,094 | 116,773 | 110,620 | 85,179 | 84,298 |
