# Supplementary material for: Kelp Culture Enhances Coastal Biogeochemical Cycles by Maintaining Bacterioplankton Richness and Regulating Its Interactions
Source: mSystems. 2023 Feb 16;8(2):e00002-23. doi: 10.1128/msystems.00002-23 (PMC10134829; doi:10.1128/msystems.00002-23)
Supplement: TABLE S2 [file msystems.00002-23-s0007.docx]

**Table S2.** Statistics of taxonomic annotation.

| **Taxonomy** | **Phylum** | **Class** | **Order** | **Family** | **Genus** | **Species** |
| --- | --- | --- | --- | --- | --- | --- |
| MW.M.1 | 17 | 27 | 75 | 126 | 198 | 51 |
| MW.M.2 | 17 | 24 | 72 | 118 | 182 | 47 |
| MW.M.3 | 16 | 25 | 76 | 126 | 195 | 47 |
| MW.M.4 | 15 | 23 | 62 | 106 | 167 | 48 |
| MW.M.5 | 11 | 19 | 59 | 108 | 196 | 60 |
| MW.M.6 | 14 | 21 | 54 | 98 | 155 | 51 |
| MW.M.7 | 11 | 19 | 56 | 96 | 170 | 48 |
| MW.M.8 | 12 | 19 | 62 | 102 | 166 | 39 |
| MW.M.9 | 14 | 20 | 51 | 82 | 136 | 41 |
| MW.M.10 | 13 | 19 | 56 | 86 | 151 | 36 |
| MW.M.11 | 13 | 21 | 61 | 101 | 163 | 41 |
| MW.M.12 | 12 | 19 | 58 | 89 | 142 | 30 |
| MW.M.13 | 13 | 21 | 61 | 110 | 174 | 63 |
| MW.M.14 | 14 | 20 | 58 | 99 | 175 | 56 |
| MW.M.15 | 12 | 19 | 63 | 110 | 182 | 45 |
| MW.M.16 | 13 | 21 | 61 | 109 | 172 | 56 |
| MW.M.17 | 9 | 18 | 59 | 100 | 152 | 39 |
| MW.M.18 | 9 | 18 | 55 | 93 | 141 | 33 |
| MW.M.19 | 12 | 22 | 64 | 101 | 148 | 29 |
| MW.M.20 | 12 | 22 | 67 | 108 | 164 | 43 |
| CW.M.1 | 16 | 23 | 65 | 101 | 162 | 33 |
| CW.M.2 | 14 | 21 | 60 | 93 | 138 | 30 |
| CW.M.3 | 11 | 19 | 52 | 84 | 107 | 23 |
| CW.M.4 | 11 | 16 | 51 | 78 | 113 | 29 |
| CW.M.5 | 12 | 19 | 65 | 104 | 138 | 32 |
| CW.M.6 | 13 | 20 | 58 | 97 | 141 | 37 |
| CW.M.7 | 11 | 15 | 51 | 88 | 145 | 39 |
| CW.M.8 | 13 | 20 | 62 | 97 | 160 | 37 |
| CW.M.9 | 16 | 25 | 66 | 110 | 167 | 32 |
| CW.M.10 | 11 | 17 | 55 | 90 | 133 | 32 |
| CW.M.11 | 13 | 19 | 55 | 83 | 101 | 15 |
| CW.M.12 | 11 | 15 | 49 | 75 | 89 | 10 |
| CW.M.13 | 12 | 18 | 59 | 88 | 128 | 28 |
| CW.M.14 | 12 | 17 | 60 | 89 | 112 | 23 |
| CW.M.15 | 11 | 17 | 59 | 99 | 128 | 25 |
| CW.M.16 | 10 | 16 | 53 | 82 | 113 | 27 |
| CW.M.17 | 12 | 19 | 59 | 102 | 183 | 54 |
| CW.M.18 | 13 | 21 | 62 | 107 | 159 | 38 |
| CW.M.19 | 14 | 21 | 60 | 98 | 152 | 30 |
| CW.M.20 | 11 | 17 | 56 | 84 | 104 | 17 |
| MW.S.1 | 12 | 15 | 49 | 69 | 91 | 18 |
| MW.S.2 | 17 | 25 | 73 | 106 | 133 | 24 |
| MW.S.3 | 14 | 20 | 63 | 92 | 129 | 31 |
| MW.S.4 | 18 | 28 | 78 | 113 | 139 | 26 |
| MW.S.5 | 22 | 33 | 93 | 136 | 169 | 27 |
| MW.S.6 | 16 | 25 | 77 | 114 | 151 | 27 |
| MW.S.7 | 16 | 26 | 78 | 107 | 137 | 25 |
| MW.S.8 | 17 | 26 | 79 | 111 | 141 | 24 |
| MW.S.9 | 15 | 22 | 75 | 107 | 148 | 26 |
| MW.S.10 | 17 | 25 | 77 | 113 | 152 | 27 |
| MW.S.11 | 18 | 28 | 81 | 112 | 153 | 23 |
| MW.S.12 | 14 | 20 | 67 | 96 | 134 | 28 |
| MW.S.13 | 16 | 23 | 68 | 93 | 123 | 21 |
| MW.S.14 | 18 | 26 | 76 | 109 | 148 | 33 |
| MW.S.15 | 15 | 23 | 72 | 105 | 139 | 30 |
| MW.S.16 | 16 | 28 | 89 | 136 | 173 | 36 |
| MW.S.17 | 18 | 26 | 78 | 106 | 141 | 27 |
| MW.S.18 | 16 | 24 | 72 | 106 | 143 | 26 |
| MW.S.19 | 19 | 29 | 85 | 120 | 158 | 23 |
| MW.S.20 | 17 | 28 | 86 | 128 | 156 | 29 |
| CW.S.1 | 24 | 34 | 99 | 140 | 182 | 42 |
| CW.S.2 | 18 | 29 | 89 | 132 | 171 | 25 |
| CW.S.3 | 19 | 30 | 92 | 133 | 157 | 22 |
| CW.S.4 | 18 | 27 | 92 | 130 | 162 | 25 |
| CW.S.5 | 17 | 26 | 95 | 138 | 176 | 26 |
| CW.S.6 | 18 | 30 | 90 | 130 | 164 | 24 |
| CW.S.7 | 15 | 25 | 81 | 112 | 150 | 24 |
| CW.S.8 | 20 | 29 | 99 | 141 | 167 | 21 |
| CW.S.9 | 16 | 25 | 90 | 130 | 167 | 25 |
| CW.S.10 | 21 | 32 | 93 | 129 | 159 | 17 |
| CW.S.11 | 20 | 32 | 91 | 131 | 158 | 25 |
| CW.S.12 | 15 | 21 | 75 | 108 | 142 | 21 |
| CW.S.13 | 21 | 31 | 95 | 134 | 167 | 18 |
| CW.S.14 | 20 | 28 | 83 | 111 | 133 | 13 |
| CW.S.15 | 22 | 32 | 99 | 142 | 175 | 25 |
| CW.S.16 | 20 | 31 | 90 | 126 | 140 | 16 |
| CW.S.17 | 22 | 34 | 103 | 153 | 189 | 28 |
| CW.S.18 | 20 | 32 | 95 | 132 | 157 | 15 |
| CW.S.19 | 20 | 31 | 89 | 128 | 153 | 22 |
| CW.S.20 | 17 | 27 | 79 | 111 | 128 | 19 |
| Total | 32 | 67 | 183 | 335 | 740 | 339 |
