# Supplementary material for: Kelp Culture Enhances Coastal Biogeochemical Cycles by Maintaining Bacterioplankton Richness and Regulating Its Interactions
Source: mSystems. 2023 Feb 16;8(2):e00002-23. doi: 10.1128/msystems.00002-23 (PMC10134829; doi:10.1128/msystems.00002-23)
Supplement: TABLE S3 [file msystems.00002-23-s0008.docx]

**Table S3.** Linear regression between alpha diversity indices of bacterioplankton and multifunctional index in different groups.

| **Stage** | **Mode** | **Alpha diversity** | **R2** | **p-value** |
| --- | --- | --- | --- | --- |
| Seedling | MW | Chao1 | **0.173** | **0.039*** |
|  |  | Shannon | -0.033 | 0.545 |
|  |  | Pielou | -0.050 | 0.757 |
|  |  | PD | 0.018 | 0.262 |
|  | CW | Chao1 | 0.054 | 0.167 |
|  |  | Shannon | 0.088 | 0.110 |
|  |  | Pielou | 0.092 | 0.105 |
|  |  | PD | 0.028 | 0.230 |
| Mature | MW | Chao1 | **0.153** | **0.049*** |
|  |  | Shannon | -0.041 | 0.618 |
|  |  | Pielou | -0.051 | 0.787 |
|  |  | PD | 0.054 | 0.166 |
|  | CW | Chao1 | -0.031 | 0.518 |
|  |  | Shannon | -0.054 | 0.885 |
|  |  | Pielou | -0.055 | 0.973 |
|  |  | PD | -0.046 | 0.686 |
